# False Localizing Trigeminal V2 Sensory Loss in Vestibular Schwannoma

**DOI:** 10.7759/cureus.9256

**Published:** 2020-07-18

**Authors:** Alejandro L Feria, Oluwaseun O Akinduro, Gazanfar Rahmathulla, Daryoush Tavanaiepour

**Affiliations:** 1 Internal Medicine, University of Kentucky, Bowling Green, USA; 2 Neurological Surgery, Mayo Clinic Florida, Jacksonville, USA; 3 Neurological Surgery, University of Florida College of Medicine, Jacksonville, USA; 4 Neurological Surgery, University of Florida Health, Jacksonville, USA

**Keywords:** false localizing sign, vestibular schwannoma, trigeminal sensory loss

## Abstract

False localizing signs involving cranial nerves are rare, even more so when involving the trigeminal nerve. Here we present the first case of trigeminal V2 sensory loss as a false localizing sign. The sensory dysfunction was caused by a large contralateral cystic vestibular schwannoma and subsequently improved after tumor resection. The clinical and radiographic features are described, and proposed mechanisms for this false localizing sign are discussed.

## Introduction

False localizing signs are neurological deficits, which deviate from what would be predicted using traditional neuroanatomical pathway and localization paradigms [1]. False localizing signs of cranial nerves, particularly the trigeminal nerve, are uncommon [2]. Although there have been five previously reported cases of lesions causing contralateral trigeminal nerve sensory dysfunction, this is the first report of a false localizing V2 trigeminal nerve sensory loss, and the first false localizing trigeminal sensory loss in the setting of a large Koos grade 4 cystic vestibular schwannoma (VS) [3-7].

## Case presentation

A 32-year-old woman with no significant past medical history presented with one month of right facial sensory loss, tinnitus, progressive imbalance, and two years of worsening hearing in the left ear. Her symptoms began with tingling on the right side of her tongue and upper lip, which progressed to her right cheek with the sensation of a swollen tongue. This continued to the point that that she would bite her right cheek when eating, and was unaware until her mouth bled. She had difficulty swallowing and felt that items frequently got stuck in her throat. She denied any nausea or vomiting, but endorsed progressively worsening headaches over the prior month.

**Figure 1:**
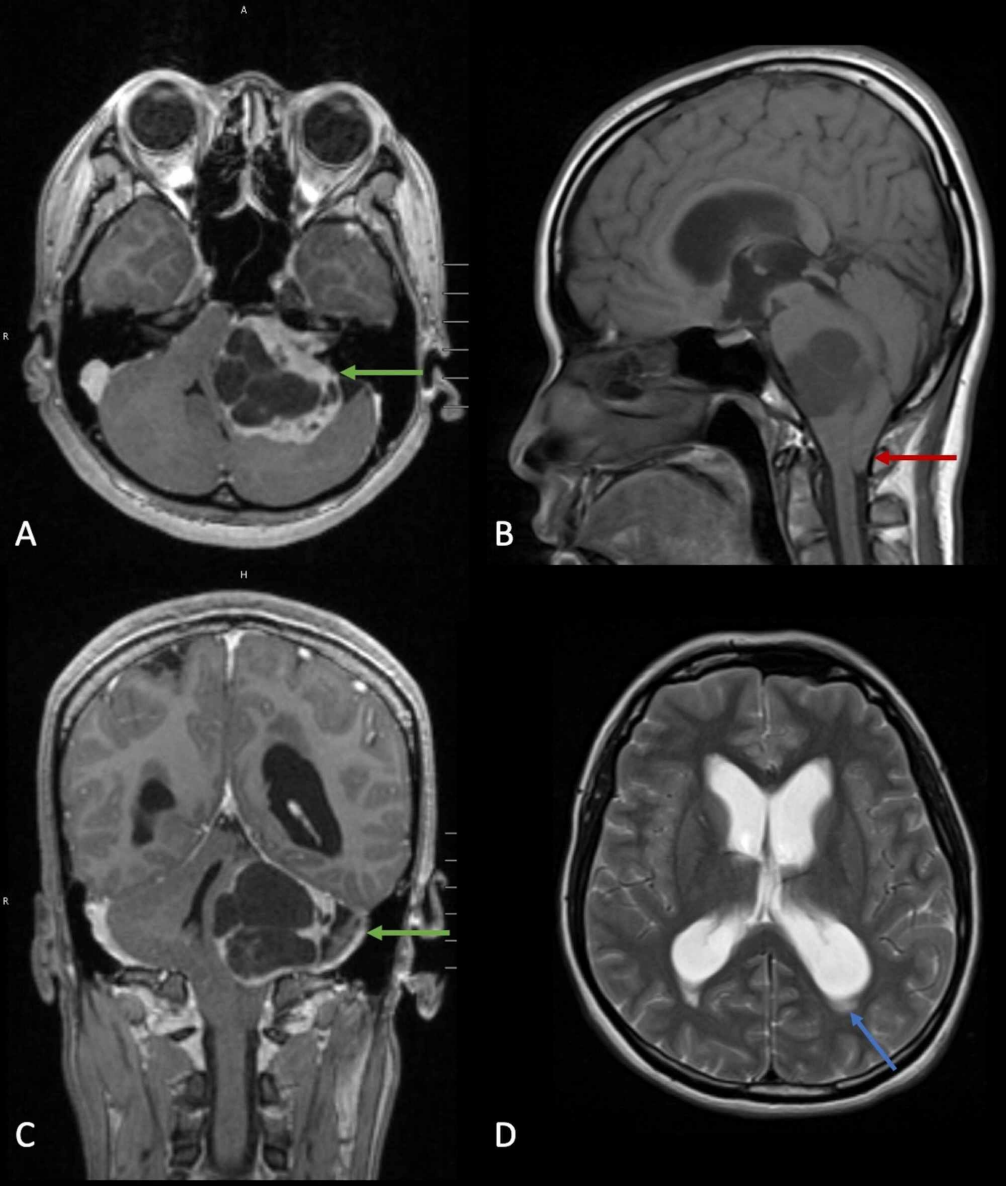
Preoperative MRI brain of large left-sided vestibular schwannoma in a patient who presented with false localizing right-sided isolated V2 sensory loss. (A) Axial T1-contrasted MRI showing a left-sided cystic heterogeneously enhancing Koos grade 4 vestibular schwannoma (green arrow). (B) Sagittal T1 MRI shows 1.7 cm tonsillar herniation (red arrow). (C) Coronal T1 contrast-enhanced MRI redemonstrates large cystic vestibular schwannoma with significant rightward brainstem displacement (green arrow). (D) Axial T2-weighted MRI shows enlarged lateral ventricles with transependymal flow (blue arrow).

On physical examination, she had loss of fine touch and pin-prick sensation in the right V2 distribution, House-Brackmann (HB) grade II facial dysfunction on the left, diminished left-sided hearing, bilateral horizontal nystagmus with fast phase to the left, right deviating uvula, loss of gag on the left, and left deviating tongue [8]. The remainder of her neurological exam was unremarkable. MRI revealed a large cystic left cerebellopontine angle tumor, measuring 4.7 × 4.8 × 4.9 cm with heterogeneous contrast enhancement and expansion of the porus acousticus consistent with a large Koos grade 4 VS (Figure 1) [9].

There was significant mass effect with severe compression of the medulla, pons, cerebellum, and fourth ventricle, herniation of cerebellar tonsils 1.7 cm below foramen magnum, and resultant obstructive hydrocephalus with T2 transependymal flow.

**Figure 2:**
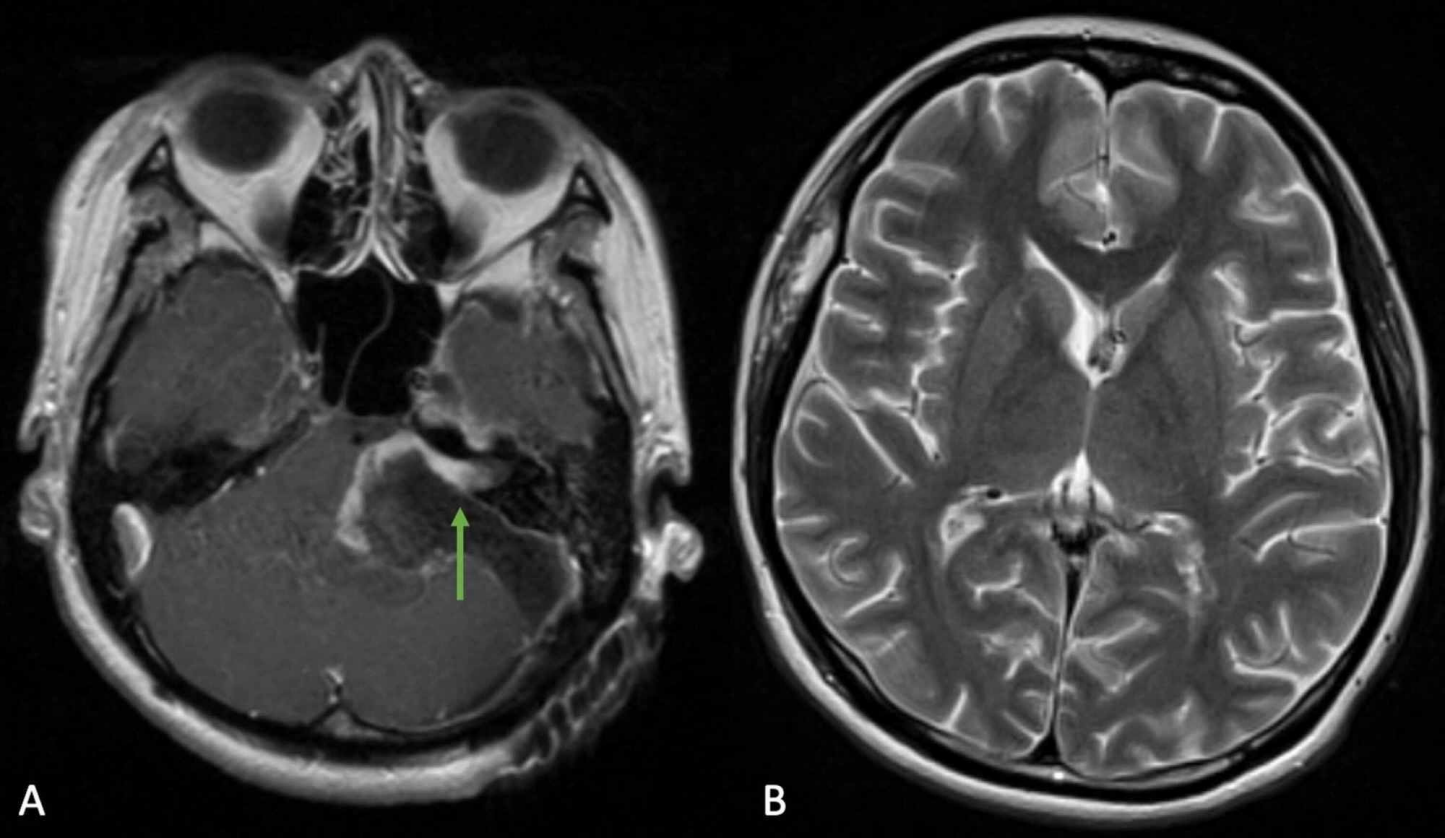
Postoperative MRI after retrosigmoid craniectomy for left-sided vestibular schwannoma resection which resulted in near total improvement of right V2 sensory dysfunction. (A) Axial T1-contrasted MRI shows resection of vestibular schwannoma with residual tumor capsule left adherent to facial nerve at the porus acousticus and improvement of rightward brainstem deviation (green arrow). (B) Axial T2 MRI shows resolution of hydrocephalus.

One day after presentation, the patient underwent placement of a right frontal ventriculoperitoneal shunt (VPS) for treatment of her hydrocephalus. Following cerebrospinal fluid (CSF) diversion, her headaches had completely resolved and there was a marked improvement in her tongue and uvula deviation. Her facial nerve function remained HB II, and the remainder of her exam was unchanged. Soon after VPS placement, she subsequently underwent a retrosigmoid craniectomy with intraoperative neuromonitoring for tumor resection. To reduce risk of debilitating facial nerve palsy, a small portion of the tumor capsule was left adherent to facial nerve at the porus acousticus (Figure 2) [10]. The tumor was confirmed to be a schwannoma.

**Figure 3:**
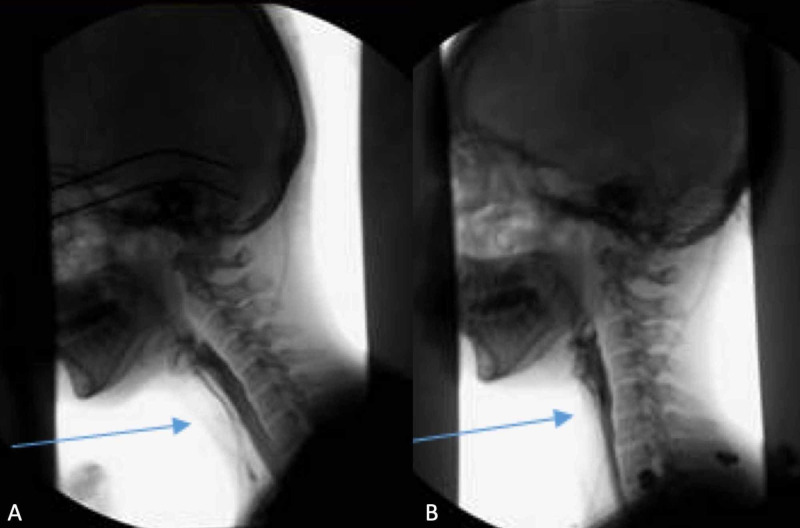
Still images from pre- and postoperative barium swallow studies. (A) Preoperative swallow study showing significant aspiration of thin and thick liquids (arrow). The patient was placed on honey thick liquid diet. (B) Postoperative swallow study after resection of vestibular schwannoma demonstrates resolution of aspiration with mild pharyngeal weakness remaining (arrow). The patient's diet was advanced to mechanical soft with thin liquids.

Postoperatively, the patient had resolution of her facial nerve palsy, now HB I, and had improvement in her contralateral sensory loss to near normal with improved fine touch and pin-prick discrimination. There was further improvement of uvula and tongue deviation as well as her swallowing function (Figure 3).

The remainder of her neurological exam was unchanged. She did remarkably well after surgery and is scheduled to undergo stereotactic radiosurgery for the residual tumor.

## Discussion

This is a rare case of contralateral V2 trigeminal sensory loss as a false localizing sign in VS. False localizing signs are classically described in the setting of a supratentorial mass causing ipsilateral hemiparesis (Kernohan’s notch phenomena) believed to be the result of brainstem herniation and displacement with resultant contralateral compression of the cerebral peduncle against the dural folds of the tentorium [11]. First described in 1904 by James Collier and subsequently expanded upon by M. Michael Gassel, there have since been multiple reports and series that describe false localizing signs involving lesions of the cerebrum, brainstem, and spinal cord [1,2].

False localizing signs involving the brainstem and cranial nerves are rare and when present they classically affect the sixth or third cranial nerves. When affecting the trigeminal nerve, false localizing signs may present as motor loss, trigeminal neuralgia, or sensory loss [12-14]. A review of the literature has identified five published cases of contralateral trigeminal sensory loss as a false localizing sign with the presented case representing the sixth reported case (Table 1) [3-7].

**Table 1:** Previously reported cases of trigeminal sensory loss as a false localizing sign.

Case	Symptoms	Tumor Location	Pathology	Treatment	Symptom Resolution
Ehni 1950 [3]	Approximately six-month history of headache and tinnitus followed by overnight onset of complete sensory loss in left V1 and V2, and moderate loss in V3 distribution. Complete paralysis of left masseter, temporalis, and pterygoid. Diminished left corneal reflex.	Supratentorial. Right pterional origin	Meningioma	Surgical resection	Complete return of sensation in all trigeminal divisions. Corneal reflex normal. No improvement in trigeminal innervated muscle paralysis.
Turnbull 1974 [4]	Nine months of headache. One week of neck pain, vomiting, slurred speech, and unsteadiness. One day of right facial weakness. Absent right corneal reflex. Right V1 sensory impairment. Right seventh nerve palsy. Partial right-sided deafness. Right ninth and tenth nerve palsies. Bilateral 11th nerve palsy. Mild right hemiparesis with mild ataxia.	Infratentorial. Large left cerebellar hemisphere cystic tumor	Cerebellar hemangioblastoma	Surgical resection	Immediate postoperative improvement of dysarthria, right facial weakness, loss of palatal sensation and right hemiparesis but absent right corneal reflex, palatal weakness, and ataxic gait persisted. Six months later, the patient showed no abnormal neurological signs.
Maurice-Williams 1975 [5]	Two years of morning vomiting with occasional occipital pain. Five months of unsteady gait, right face and palate numbness, transient diplopia, blurred vision, and dysphagia. Loss of pin-prick and light touch on right V1, V2, and V3. Absent right corneal reflex. Right 9th and 10th nerve dysfunction. Right leg cerebellar ataxia	Infratentorial. Left inferior leaf of lateral tentorium just above most outer part of petrous temporal bone	Meningioma	Surgical resection	At six weeks postop, all symptoms resolved except persistent reduction of the right corneal reflex and slight dulling of right oropharyngeal sensation.
Koenig et al. 1984 [6]	Left ear tinnitus of two years with left-sided deafness of eight months. Four months of progressive loss of right facial sensation. Decreased right corneal reflex. Decreased sensation, hypesthesia, hypoalgesia of right V1, V2, and V3 (motor normal). Orbicularis oculi reflex study showed absence of early and late response on the right of right-sided supraorbital nerve stimulation.	Infratentorial. Left cerebellopontine angle	Vestibular schwannoma	Surgical resection	One week postop, studies showed normal early and late responses from right orbicularis oculi muscle on stimulation of right supraorbital nerve. Other symptoms not reported.
Ro et al. 1995 [7]	Six years of progressive right facial weakness, three years of right facial numbness and diplopia, one year of unsteadiness and dysphagia. Right trigeminal sensory disturbance in V1 and V2. Depressed corneal reflexes R>L. Right sixth and seventh nerve palsies. Left hearing impairment. Weakness of the left soft palate, generalized hyperreflexia, and a left extensor plantar reflex. Masseter strength was normal.	Infratentorial. Left cerebellopontine angle	Vestibular schwannoma	Surgical resection	Three days postop showed improved diplopia, right facial hypoalgesia, and swallowing dysfunction. Right facial palsy persisted.

This case is unique in the following respects: it is the first to describe a false localizing trigeminal sensory loss isolated to the V2 distribution, and the first in the setting of a large cystic Koos grade 4 VS.

**Figure 4:**
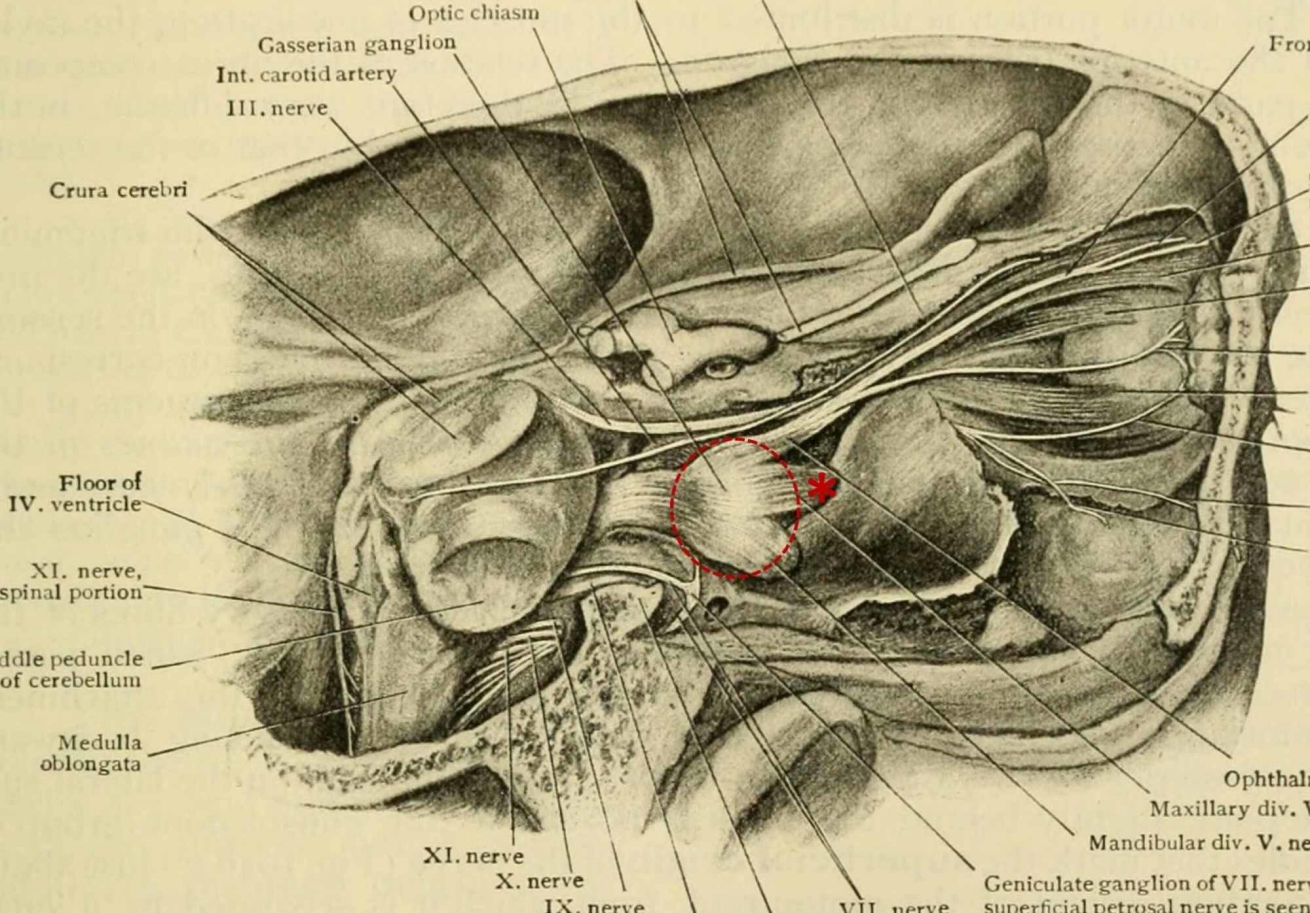
Anatomic illustration of trigeminal nerve course and sites of proposed nerve disruption at foramen rotundum or trigeminal ganglion. Anatomic illustration of normal right trigeminal nerve course with emphasis placed on proposed compressive sites resulting in selective contralateral V2 sensory loss in the presented case: 1. (red asterisk) V2 division (maxillary nerve) as it enters foramen rotundum and 2. (dashed circle) preferential compression of maxillary nerve fibers in the trigeminal (Gasserian) ganglion. Reprinted from ‘Human Anatomy: Including Structure and Development and Practical Considerations’ [20].

There have been multiple proposed mechanisms for contralateral trigeminal nerve dysfunction, including sheer and torsional forces applied to nerve rootlets, foramen, and other compressive zones as a result of mass effect and brainstem displacement, compressive forces from arachnoid bands, or vascular compression at the nerve root entry zone [14-16]. In the presented case, the patient had multiple ipsilateral cranial nerve palsies of the facial, vestibulocochlear, glossopharyngeal, vagal, and hypoglossal nerves, which were likely due to direct mass effect from the tumor. We propose two potential theories for the contralateral sensory loss being limited to the V2 distribution. First, many studies have shown that there is a somatotropic organization within the Gasserian ganglion, with ophthalmic fibers located anteromedial, mandibular located posterolateral, and maxillary fibers in the middle [17-19]. It is possible that the organization of these fibers persists, and slightly varies within the trigeminal nerve and nuclei. This would leave potential for preferential compression of fibers in a particular distribution. Second, it is possible that there was some element of compressive, torsional, or sheer force applied to the nerve as it enters foramen rotundum (Figure 4) [20].

Of note, the right-sided V2 sensory loss improved after tumor debulking and resultant improvement of her brainstem displacement and not after VPS and treatment of her intracranial hypertension, which did provide some improvement to her ipsilateral glossopharyngeal and hypoglossal palsies. In this light, the cause of the sensory loss was more likely due to direct compressive forces from the tumor and significant brainstem displacement rather than elevated intracranial pressure.

## Conclusions

We have presented the sixth reported case of a false localizing contralateral trigeminal sensory loss and the first such case to be restricted to the V2 distribution. The recognition of false localizing findings in the neurological exam is important to improve diagnostic accuracy. The mechanisms causing false localizing signs remain poorly understood.

## References

[1] Gassel MM (1961). False localizing signs. A review of the concept and analysis of the occurrence in 250 cases of intracranial meningioma. Arch Neurol.

[2] Larner AJ (2003). False localising signs. J Neurol Neurosurg Psychiatry.

[3] Ehni G (1950). ‘False’ localizing signs in intracranial tumor; report of a patient with left trigeminal palsy due to right temporal meningioma. AMA Arch Neurol Psychiatry.

[4] Turnbull AR (1974). Multiple false localizing signs in intracranial tumor. Case report. J Neurosurg.

[5] Maurice-Williams RS (1975). Multiple crossed false localizing signs in a posterior fossa tumour. J Neurol Neurosurg Psychiatry.

[6] Koenig M, Kalyan-Raman K, Sureka ON (1984). Contralateral trigeminal nerve dysfunction as a false localizing sign in acoustic neuroma: a clinical and electrophysiological study. Neurosurgery.

[7] Ro LS, Chen ST, Tang LM, Wei KC (1995). Concurrent trigeminal, abducens, and facial nerve palsies presenting as false localizing signs: case report. Neurosurgery.

[8] House JW, Brackmann DE (1985). Facial nerve grading system. Otolaryngol Head Neck Surg.

[9] Erickson NJ, Schmalz PGR, Agee BS, Fort M, Walters BC, McGrew BM, Fisher WS (2019). Koos classification of vestibular schwannomas: a reliability study. Neurosurgery.

[10] Akinduro OO, Lundy LB, Quinones-Hinojosa A, Lu VM, Trifiletti DM, Gupta V, Wharen RE (2019). Outcomes of large vestibular schwannomas following subtotal resection: early post-operative volume regression and facial nerve function. J Neurooncol.

[11] Zhang CH, DeSouza RM, Kho JSB, Vundavalli S, Critchley G (2017). Kernohan-Woltman notch phenomenon: a review article. Br J Neurosurg.

[12] Grigoryan YA, Onopchenko CV (1999). Persistent trigeminal neuralgia after removal of contralateral posterior cranial fossa tumor. Report of two cases. Surg Neurol.

[13] Kondoh T, Tamaki N, Takeda N, Shirataki K, Mastumoto S (1989). Contralateral trigeminal neuralgia as a false localizing sign in calcified chronic subdural hematoma: a case report. Surg Neurol.

[14] Matsuura N, Kondo A (1996). Trigeminal neuralgia and hemifacial spasm as false localizing signs in patients with a contralateral mass of the posterior cranial fossa. Report of three cases. J Neurosurg.

[15] Paillas JE, Pellet W, Janny P, Tournilhac M, Komminoth J (1969). Contralateral involvement of cranial nerves in posterior cranial fossa tumors. (Article in French). Rev Neurol.

[16] Michelucci R, Tassinari CA, Plasmati R, Rubboli G, Forti A, Tognetti F, Calbucci F (1989). Trigeminal neuralgia associated with contralateral intracranial tumour: a false localising sign caused by vascular compression? Report of two cases. J Neurol Neurosurg Psychiatry.

[17] Chai Y, Chen M, Zhang W, Zhang W (2014). Somatotopic organization of trigeminal ganglion: three-dimensional reconstruction of three divisions. J Craniofac Surg.

[18] Joo W, Yoshioka F, Funaki T, Mizokami K, Rhoton AL (2014). Microsurgical anatomy of the trigeminal nerve. Clin Anat.

[19] Wei W, Han Z, Chen M, Zhang W, Chai Y, Wang Y, Zhang W (2017). Three-dimensional reconstruction of the distribution of neurons contributing to ophthalmic, maxillary, and mandibular nerves in the trigeminal ganglion of experimental model. J Craniofac Surg.

[20] Dwight T, Hamann C, McMurrich J, Piersol G, White J (1923). Human Anatomy: Including Structure and Development and Practical Considerations (Eight Edition). Fig. 1053.

